# Supplementary material for: Optimization of technique parameters of pneumatic molding for rice straw bowl tray
Source: PLoS One. 2022 Sep 6;17(9):e0270421. doi: 10.1371/journal.pone.0270421 (PMC9447898; doi:10.1371/journal.pone.0270421)
Supplement: Attachment — Submitted filename: Response to Reviewers.pdf [file pone.0270421.s002.pdf]

Response to Reviewers

Dear editor,·

Thank you for your useful comments and suggestions on thelanguage and structure of our manuscript. We have modified themanuscript accordingly, and detailed corrections are listed below point by point:

(1) It appears that your ORCiD iD has not been validated in your Editorial Manager account and we are unable to proceed until that step is complete.

Response： We have completed the registration work for ORCiD.

(2) We notice that your manuscript file was uploaded on June 23, 2021. Please can you upload the latest version of your revised manuscript as the main article file, ensuring that does not contain any tracked changes or highlighting. This will be used in the production process if your manuscript is accepted. Please follow this link for more information: http://blogs.PLOS.org/everyone/2011/05/10/how-to-submit-your-revised-manuscript/

Response： We have removed the original manuscript and a amended manuscript without any tracked changes or highlighting wasuploaded.

(3) Thank you for stating the following financial disclosure: "This work is

supported jointly byHainan natural science foundation-The agglomerate structure of red soil in Hainan province was improved by conservation tillage（519QN289）;Central Public-interest Scientific Institution Basal Research Fund for Chinese Academy of Tropical Agricultural Sciences（No.19CXTD-31）;2020 national subsidization of basic agricultural technology(18200059)." Please state what role the funders took in the study. If the funders had no role, please state: "The funders had no role in study design, data collection and analysis, decision to publish, or preparation of the manuscript."If this statement is not correct you must amend it as needed. Please include this amended Role of Funder statement in your cover letter; we will change the online submission form on your behalf.

Response： The funder is the projects leader and is responsible for the formulation and implementation of the overall project plan.

(4) Please amend the title either on the online submission form or in your manuscript so that they are identical.

Response: The title has been amendied to ensure that it is identical on the online submission form or in manuscript.

(5) Can you please upload an additional copy of your revised manuscript that does not contain any tracked changes or highlighting as your main

article file. This will be used in the production process if your manuscript is accepted. Please amend the file type for the file showing your changes to Revised Manuscript w/tracked changes. Please follow this link for more information: http://blogs.PLOS.org/everyone/2011/05/10/how-to-submit-your-revised-manuscript/

Response： Two documents "Revised Article with Changes Highlighted" and "Manuscript" were uploaded. "Revised Article with Changes Highlighted" showing the changes we have made since the original submission. "Manuscript" does not show any changes.

(6) Please ensure that you refer to Figure 10 and 11 in your text as, if accepted, production will need this reference to link the reader to the figure.

Response： In this paper, Figure 10 was applied when analyzing the interactions of vacuum degree and adsorption time versus rupture-resisting strength of straw bowl as show in line 296, and figure 11 was cited in the presentation of the straw bowl as show in line 327.

(7) Please include a copy of Table 1 which you refer to in your text on page 4.

Response： Documents that include all the tables in manuscript has been

uploaded.

(8) We note that your author list was updated during the revision process. Please complete our Authorship Change Form by following this link:http://journals.plos.org/plosone/s/file?id=13d0/plos-one-change-to-authorship-form.docx. If you are adding or removing more than 2 authors, you can complete multiple forms and submit as many forms as needed to reflect the updates. Please return the form(s) as an attachment by emailing plosone@plos.org or by uploading it as a submission file labeled with the file type ‘Other’. Please note that if your manuscript is accepted, we will not be able to complete the publication process without the completed form.

Response：Authorship Change Form has been completed and uploaded.

(9) Please include a separate caption for each figure in your manuscript.

Response：We have been include a separate caption for each figure in manuscript.

(10) Please include captions for your Supporting Information files at the end of your manuscript, and update any in-text citations to match accordingly. Please see our Supporting Information

Response：Captions in-text has been changed to ensure that matches the content in supporting information.
