# Supplementary material for: Optimization of technique parameters of pneumatic molding for rice straw bowl tray
Source: PLoS One. 2022 Sep 6;17(9):e0270421. doi: 10.1371/journal.pone.0270421 (PMC9447898; doi:10.1371/journal.pone.0270421)
Supplement: Attachment — Submitted filename: Response to Reviewers.docx [file pone.0270421.s003.docx]

**Response to Reviewers**

Dear editor,·

Thank you for your useful comments and suggestions on thelanguage and structure of our manuscript. We have modified themanuscript accordingly, and detailed corrections are listed below point by point:

（1） In the state-of-the-art section you mainly explain the raw material used by other research groups to prepare the seedling bowl, but you scarcely report quantitative results in the existing bowls performance with respect to the parameters you decided to take into consideration. Moreover, you should highlight if any other study considered the same or similar quantitative performance parameters.

**Response**：In this paper, the research of biomass seedling bowl molding technology and molding process are discussed, and the research status of pneumatic forming technology is explained.

（2） Explain what you mean by straw quality score.

**Response**：The explanation of straw quality score appears in the first paragraph of “2.3.1 Production method of potted tray”.

（3） In the text should emerge before why you chose to evaluate the vacuum degree, pressure preservation time and adsorption time factors and why they are so relevant during the molding process. Try to briefly explain the concept when you first introduce the factors you decided to evaluate.

**Response**：The reasons for selecting vacuum degree, adsorption time and pressure preservation time as experimental factors and their important correlation to the forming effect of seedling tray are described in the first three sections of “2.3.2 test factors”.

（4） It is not clear what you mean by bowl forming rate.

**Response**：Bowl forming rate is the bowl hole molding rate, the mean of bowl hole molding rate and the calculation formula are detailed in the second paragraph of “2.3.3 Test indices and measurement method”.

（5） In the response surface test paragraph, you should insert a few lines at the beginning of the paragraph to introduce the reader to what this part of the paper is about, what tests will be explained in the paragraph and what parameters change from one test to the other.

**Response**：The main contents of response surface analysis have been introduced in the first paragraph of “3.2 Response surface analysis”.

（6） In the response surface test paragraph, what is the software used to obtain the model shown in the figures?

**Response**：The software used for response surface analysis has been described in the first paragraph of “3.2 Response surface analysis”.

（7） Define in the text the acronym ANOVA before using it.

**Response**：ANOVA has been replaced with variance analysis.

（8）A few expamples of unclear senstences/ misused English terms:

- Line 3: light quality.

- Line 3: The commonly used plastic plate mostly made of polyethylene, with low price, light quality and good water protection, but transplanting mold damage root system, not easy to degradation, improper recycling can cause environmental pollution.

- “..forming ways, not only improve the utilization rate of agricultural waste, give full play to the residual value of agricultural waste, but also solve the agricultural waste and environmental pollution.”

**Response**：The problems of unclear senstences/ misused English terms were revised.

（9） Be sure each sentence makes sense. Make shorter sentences if needed to prevent the loss of the key concept you want to express by the sentence.

**Response**：Inappropriate sentences in the text have been corrected.
